#  The Effects of Valerian Root on Hot Flashes in Menopausal Women 

**Published:** 2013

**Authors:** Parvaneh Mirabi, Faraz Mojab

**Affiliations:** a ***Department of Midwifery, Zanjan Branch, Islamic Azad University, Zanjan, Iran.***; b ***Department of Pharmacognosy, School of Pharmacy, Shahid Beheshti University of Medical Sciences, Tehran, Iran. ***; c ***Pharmaceutical Sciences Research Center, Shahid Beheshti University of Medical Sciences, Tehran, Iran. ***

**Keywords:** Valerian, Hot flash, Menopause, Herbal medicine

## Abstract

Hot flash is among the most common complaints of menopausal women, affecting their career, social activities and quality of life. This study aimed to investigate the effects of Valerian on hot flashes in menopausal women. In this double blind clinical trial, 68 menopausal women with the chief complaint of hot flash were enrolled using sampling at hand and were randomly divided into drug and placebo groups. The women in the drug group were prescribed 255 mg Valerian capsules 3 times a day for 8 weeks. The women in the placebo group were prescribed identical capsules filled with starch. Then, severity and frequency of hot flashes were measured and recorded through questionnaires and information forms in three levels (2 weeks before, four and eight weeks after the treatment). The Severity of hot flashes revealed a meaningful statistical difference pre- and post- Valerian treatment (p <0.001) while this difference was not meaningful in the placebo group. Further, the comparison of the two groups regarding the severity of hot flash after the treatment showed a meaningful statistical difference (p <0.001). Valerian has also led to a reduction of hot flash frequencies 4 and 8 weeks after the treatment (p <0.001) but this difference was not meaningful in drug like group. Valerian can be effective in treatment of menopausal hot flash and that it can be considered as a treatment of choice for reduction of hot flashes among the women who are reluctant to receive hormone therapy due to fear or any other reason.

## Introduction

Women, as the key members of society who constitute more than half the population, live longer than men. They spend 30 years of their lives after menopause, which is more than one-third of their lives (1). 

Forty percent of premenopausal women, 70 to 80 percent of women who naturally go through menopause and 90 to 100 percent of those who have had ovarectomy, experience hot flashes. It occurs more than 10 times a day in 30 percent of them (2, 3). Hot flash and perspiration do not jeopardize one’s life but they may result in anxiety and discomfort and they can even affect one’s career, housework and leisure time (4). 

Hot flashes usually occur during the night so they affect sleeping patterns and lead to perspiration and sleeplessness (5). Definitely, insomnia causes anger, restlessness and reduces mental functions, hence makes the body vulnerable to all sorts of stress disorders and can be indirectly related to coronary heart diseases (6).

The proposed treatments are not acceptable for women or practitioners due to their side effects, inappropriate efficacy, or unclear dosage. Furthermore, menopause symptoms are different among populations with different diets, especially those rich in phytoestrogen, which has attracted the attention of women and practitioners to natural, inexpensive herbs that bear little or no side effect. Herbs with estrogen-like components such as black cohosh, kava kava, gingko, ginseng and valerian are recommended for the reduction of menopause symptoms (7). Previously, some researches were done on many herbs with relief and recurrence of menopausal hot flashes, *e.g. *avocado and soybean oil (piascledine^®^) (8), licorice (9) and *Pimpinella anisum *(10).

Valerian is a grass, whose root and rhizome are used for this purpose. The name “valerian” is derived from the term “valer” that means nice and healthy (11). Its root is used for the treatment of different diseases such as dizziness, neural pains, neural unilateral headaches and anxiety. Because of its phytoestrogenic components, valerian is also recommended for the reduction of menopause symptoms, sleeplessness and mental disorders (7, 12, 13).

The effects of Valerian on menopause hot flashes are examined in this study since the plant is easily available in Iran. In case of achieving suitable effectiveness, the results would be reported to health care centers, especially obstetricians as health care observers, in order to improve the quality of women’s lives.

## Experimental

This study was a randomized double blind clinical trial. Data were collected in three stages (before the treatment, 2 weeks and 4 weeks after the treatment) and from two groups including valerian and placebo group. The subjects include 68 women between the ages of 45 to 55, who referred to health care centers in Zanjan, with a chief complaint of hot flashes and willing to be treated. The subjects were enrolled using sampling at hand and were randomly divided into two groups.

The inclusion criteria are as follows:

I. Being literate or having a literate person at home.

II. Being a menopausal woman between the ages of 45 and 55.

III. Elapse of at least one and at most 5 years after menopause.

IV. Not having known physical or mental diseases.

V. Not using relaxation techniques and hormonal or herbal therapies.

VI. Not using cigarette and alcohol. 

VII. Not having stressors during the past 6 months.

VIII. Not having a family history of estrogen-related cancer.

Before entering the study, all subjects were given information about the research methodology and entered the project after signing the informed written consent form. They were also asked to record any problems like headache, nausea, gastrointestinal disorders, anorexia and any other problem during both stages of treatment in special forms. Severe symptoms had to be reported to the researcher.

Subject using special medications, having mental, endocrine and gynecological disorders, or being treated with hormone replacement therapy, experiencing abnormal menopause caused by irradiation and being allergic to Valerian were excluded from the study.

Data were collected using questionnaires filled by the researcher and two other information forms. At first, questionnaire number 1 (Table 1) was filled with information about demographics, obstetrics history and questions about doing sports, severity of hot flashes and the manner they were treated. Then, during the two weeks before treatment, information forms (1 and 2) were filled with study diet, frequency and severity of hot flashes. Reliability and validity of questionnaires were approved by content validity and re-tested with the correlation of 90%.

Valerian was purchased as bulk from Zardband Company, validated by one of the authors (Dr. Mojab). Afterward, valerian was ground, and then filled in capsules using an automatic capsule filling machine. The placebo was made using the same machine but capsules were filled with potato starch.

**Table 1:** A comparison between valerian and placebo subjects’ characteristics.

		**Treatment Categories**	
	**Variable**	**Valerian**	**Placebo**
Age		51.2 ± 2.6	51.7 ± 2.9
Age at menarch		13.17 ± 1.34	13.30 ± 1.06
Age at menopause		49.30 ± 3.01	48.90 ± 2.72
BMI		28.54 ± 3.01	27.8 ± 3.82

Capsules containing 225 mg of valerian root were prescribed 3 times a day for eight weeks for the case group. The placebo group received the same dosage of the placebo. Encoded capsules were given to the subjects in both groups in similar packages. Neither the patients nor the researchers were aware of the assigned groups.

Information forms (1 and 2) were given to research centers and received by subjects from two weeks before the program until the end of the study in order to determine the severity and frequency of hot flashes and daily meals. The data were collected and analyzed at the end of each week.

SPSS version 16 was used for data analysis in this study. Descriptive statistics including distribution tables, mean and standard deviation were used to describe subjects’ characteristics. As the response variable was graded and data were not natural, at first, the severity of hot flashes was compared in 3 cycles using the Friedman test. Then, responses between the two groups were compared using Man-Whitney test. Once Freidman test was meaningful, treatment cycles were two by two compared using corrected Alpha and Wilcoxon test.

## Results

From 76 patients who were equally divided into two groups, 5 subjects from placebo and 3 from valerian groups were excluded from the study due to the reasons such as irregular use as a result of forgetting, inefficacy, inaccurate recording, gastrointestinal disorders, dizziness and taking other medications affecting hot flashes. Therefore, the data was collected from 68 subjects.

As shown in Table 1, there was no meaningful difference between the two groups with regard to age, age at menopause and BMI. Subjects were matched for education, occupation, socioeconomic status and there was no meaningful difference between the two groups. Most research centers that finished the study in the drug group were satisfied with valerian, *i.e*., valerian significantly outperformed the (p < 0.001). Fisher’s exact test showed no meaningful difference in side effects between valerian and placebo (p > 0.005).

Based on Mann-Whitney test, no meaningful difference was observed in severity of hot flashes before the treatment (p > 0.005). However, 4 and 8 weeks after the treatment, this variable showed a meaningful difference between the two groups (p < 0.001).

Friedman and Wilcoxon tests (with corrected Alpha) were used to compare the severity of hot flashes in both groups before and after the study and a meaningful difference was observed in Valerian group (p < 0.001) but this difference was not statistically meaningful in placebo group before and after the study (Table 2).

**Table 2:** Comparison of mean severity of hot flashes in both groups.

	**Mean and Standard Deviation**			
**Treatment rounds**	**Before treatment**	**4 weeks after treatment**	**8 weeks after treatment**	p-value**
**groups**				
Valerian	9.82 ± 1.87	7.01 ± 1.86	5.23 ± 1/52	p < 0.001
Placebo	9.96 ± 1.84	9.60 ± 1.76	9.86 ± 1.95	NS
p-value*	NS	p < 0.001	p < 0.001	

*Man-Whitney U test

** Friedman &Wilcoxon

Comparing the mean frequency of hot flashes in each group, results of Wilcoxon test showed that there is a statistically meaningful difference in Valerian group, 4 and 8 weeks after the treatment (p < 0.001) but such a difference was not observed in placebo group within the same period (Table 3).

**Table 3:** Comparison of mean frequency of hot flashes in both groups

**Mean and Standard Deviation **				
**Treatment rounds**	**Before treatment**	**4 weeks after treatment**	**8 weeks after treatment**	**p-value **
**groups**				
Valerian	7.91 ± 30.0	5.62 ± 0.46	4.83 ± 0.52	p < 0.001
Placebo	7.73 ± 42.0	7.40 ± 0.29	7.75 ± 0.32	NS
p-value*	NS	p < 0.002	p < 0.001	

## Discussion

The global approach toward herbal treatment is very complicated and includes different factors in different areas. Safety, availability, treatment control and consistency with holistic medicine that underscores natural products may be among the most important factors in this approach. Certainly, the negative effects of chemical medications, lack of their efficiency in some cases and their side effects are among other influencing factors.

There are 7500 herbs in Iran, 1300 of which are endemic. Valerian, borage, chamomile, marjoram, *melise*, ginger, *Cotoneaster frigidus *and manna are among the most popular herbs (14).

Valerian is a phytoestrogenic herb that contains volatile oils including monoterpenes, sesquiterpenes and valepotriates beside the components resulted from their breakdown (15, 16).

Phytoestrogens are estrogen-like complexes that can be found in plants and have estrogenic and antiestrogenic qualities. There are strong evidences supporting the effects of these herbs on hot flash and other menopausal symptoms. They also reduce the risk of heart diseases and osteoporosis, alleviate the menopause symptoms especially hot flashes, and improve memory and sleeping patterns. Phytoestrogens act as an estrogen agonist and may show estrogen-like effects (17).

Steroid strength of phytoestrogens is estimated from 1/50 to 1/2000 steroids, but they don’t have side effects like endometrial neoplasm and breast cancer as they make body produce hormones (18).

In this study, valerian phytoestrogenic capsules have reduced the frequency and severity of hot flashes. This reduction was meaningful as compared with those before the treatment. Such a difference was not observed in placebo group, though. The above-mentioned findings were consistent with those of a similar study performed by Kazemian (19). Of course, the effect of valerian drug on hot flash was studied by Kazemian while we used its root powder.

The results of different studies have proved the efficacy of the placebo on menopause symptoms especially hot flashes. Furthermore, in a study that was performed to examine the effects of Fluoxetine on hot flash, it was proved that both Fluoxetine and placebo lead to a reduction in frequency and severity of hot flash but Fluoxetine was much significantly more effective than placebo (20).

The result of Baghdari study (88) showed no meaningful difference between linen seed powder and placebo in reducing the frequency of hot flashes (21).

Besides, in a randomized study performed on women taking estrogen implants in England, no difference was observed between women who took drug and those who took placebo from physiological and physical aspects (22)


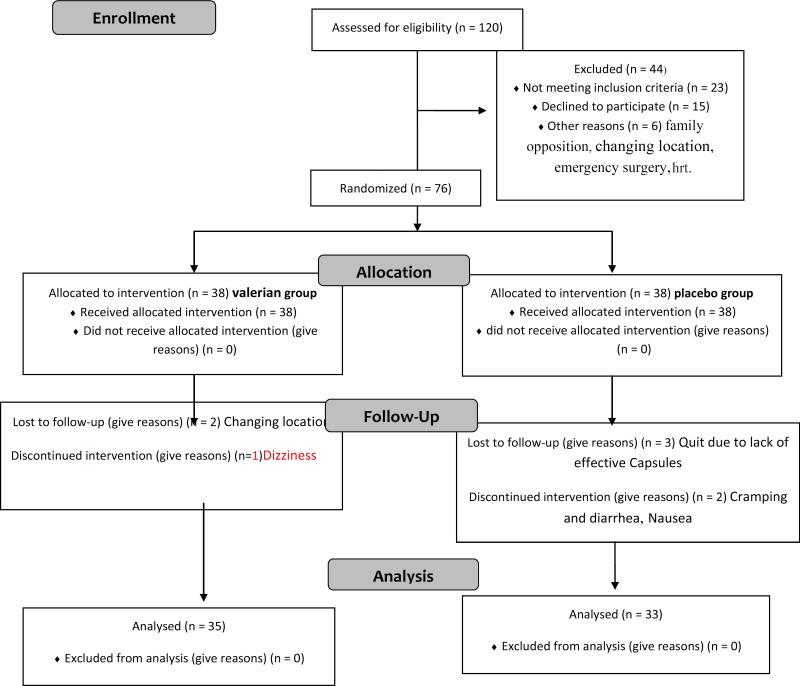


However, subjects in our study did not respond to placebo. The results of Frei-Kleiner study also showed that the effect of tested drug (phytoestrogen) on menopause symptoms among perimenopausal women was more meaningful than among women who were menopausal. This can justify the results of our study as all of our subjects were menopausal (23). In order to control the effect and intervention of the foods containing phytoestrogen, subjects were asked to record their daily meals and they were excluded from the study in case of taking those foods. As a result, it can be strongly stated that the reduction in severity and frequency of hot flashes is merely due to the phytoestrogen in valerian, which can be prescribed to women who suffer from hot flashes in a simple and non-invasive manner.

Due to the controversial effects of phytoestrogen as compared to hormone replacement therapy, long term studies and possible relapses are recommended.
